# Characterisation of the changing genomic landscape of metastatic melanoma using cell free DNA

**DOI:** 10.1038/s41525-017-0030-7

**Published:** 2017-09-04

**Authors:** Anthony Cutts, Oliver Venn, Alexander Dilthey, Avinash Gupta, Dimitris Vavoulis, Helene Dreau, Mark Middleton, Gil McVean, Jenny C. Taylor, Anna Schuh

**Affiliations:** 1 Nuffield Division of Clinical Laboratory Sciences (NDCLS), Oxford Molecular Diagnostics Centre, University of Oxford, Oxford, UK; 2 NIHR Oxford Biomedical Research Centre, Oxford, UK; 3 Lighthouse Cancer Diagnostics Ltd, Oxford, UK; 4 The Wellcome Trust Centre for Human Genetics, University of Oxford, Oxford, UK; 5 University of Oxford Department of Oncology, Churchill Hospital, Oxford, UK; 6 Department of Haematology, Oxford University Hospital Trust, Oxford, UK; 7 Present Address: NHGRI-NIH, 20892 Bethesda, MD USA; 8 Present Address: Department of Medical Oncology, The Christie NHS Foundation Trust, Manchester, UK

## Abstract

Cancer is characterised by complex somatically acquired genetic aberrations that manifest as intra-tumour and inter-tumour genetic heterogeneity and can lead to treatment resistance. In this case study, we characterise the genome-wide somatic mutation dynamics in a metastatic melanoma patient during therapy using low-input (50 ng) PCR-free whole genome sequencing of cell-free DNA from pre-treatment and post-relapse blood samples. We identify de novo tumour-specific somatic mutations from cell-free DNA, while the sequence context of single nucleotide variants showed the characteristic UV-damage mutation signature of melanoma. To investigate the behaviour of individual somatic mutations during proto-oncogene B-Raf -targeted and immune checkpoint inhibition, amplicon-based deep sequencing was used to verify and track frequencies of 212 single nucleotide variants at 10 distinct time points over 13 months of treatment. Under checkpoint inhibition therapy, we observed an increase in mutant allele frequencies indicating progression on therapy 88 days before clinical determination of non-response positron emission tomogrophy-computed tomography. We also revealed mutations from whole genome sequencing of cell-free DNA that were not present in the tissue biopsy, but that later contributed to relapse. Our findings have potential clinical applications where high quality tumour-tissue derived DNA is not available.

## Melanoma: Whole Genome Sequencing of Liquid biopsy without PCR amplification

A blood-based test can find mutations in minute amounts of circulating tumour (ct) DNA to help guide treatment decisions. Anna Schuh and colleagues from the University of Oxford, UK, developed a protocol for picking up low levels of DNA shed by tumours into the bloodstream and then, without using an amplification step that introduces biases, sequence the entire genome. The researchers used this technique to identify mutations that were then tracked across 10 different time points for one patient undergoing treatment for melanoma. They identified exposure to UV-light as the source of the mutation that caused the skin cancer. Their test detected genetic signs of relapse 3 months before scans did and revealed additional mutations not found in the tissue biopsy sample taken. The findings support the use of such a test in clinical care.

## Introduction

Whole exome sequencing (WES) and whole genome sequencing (WGS) of DNA derived from tumour biopsies has generated atlases of somatic mutations across cancer types, revealing deep genetic heterogeneity within and between tumours.^1–3^ These catalogues have improved the molecular classification of cancer types and are a resource for drug development.^4, 5^ However, for WES and WGS, high quality DNA from fresh tumour material is required, which has limited their widespread application in clinical practice.^6–8^ Establishing the acquired genetic profile of a cancer by using targeted sequencing of specific cancer genes is increasingly used to both help diagnosis and guide treatment decisions and commonly uses formalin fixed paraffin-embedded tumour material. However, tumour sequencing has its limitations: biopsies are invasive limiting treatment monitoring, obtaining tumour material can be difficult and provides only limited representations of the potential underlying heterogeneity through inherent sampling bias. In the era of molecularly targeted high-cost continuous maintenance therapy, monitoring of treatment response is essential to avoid continuing ineffective therapies, to prevent unnecessary side effects from inappropriate therapy, and to determine the benefit of new therapeutics as early as possible in the treatment course. Therefore, there is an urgent need for new approaches to comprehensively and non-invasively identify and track changes in the mutational landscape across primary tumours and metastases.

Cell-free DNA (CfDNA) molecules are short nuclear DNA fragments present in several bodily fluids generated predominantly through cell apoptosis.^9^ The abundance of cfDNA in blood plasma is highly variable and is influenced by a number of covariates, including post blood draw contamination.^10^ The average length of cfDNA are 167 bases with a periodicity of 10 bases, with increasing evidence suggesting the majority of tumour fragments may be shorter than non-tumour derived cfDNA.^11–13^ In cancer patients, tumour-originated circulating tumour DNA (ctDNA) typically constitute a minority population of cfDNA in plasma.^14^ Observed correlations between ctDNA abundance and imaged tumour size,^15^ stage and type^14^ suggest a potential molecular representation of disease. To address the challenges of low ctDNA abundance high efficiency and high fidelity detection approaches are required for which various digital PCR and targeted next generation sequencing (﻿NGS) approaches have been developed.^15–18^ Additionally, WES studies have been used to identify low level variants and monitor tumour evolution.^19–22^ So far, genome-wide sequencing efforts of cfDNA have focussed on detection of a limited set of tumour specific mutations in patients with advanced cancers.^21, 23–27^

In the current study we tracked mutations from pre-treatment, post-treatment and relapse cfDNA samples from a patient with malignant melanoma across 10 different time points. We developed a low-input PCR-free protocol and bioinformatics pipeline to facilitate WGS analysis of cfDNA. We provide proof-of-principle that this approach is feasible and reliably identifies a broad spectrum of different types of mutations including those in the non-coding regions of the genome. Finally, we identify mutation signatures associated with exposure to UV-light from cfDNA and identify clinically actionable mutations that can be used to direct treatment and molecular monitoring of treatment response.

## Results

### WGS sequencing quality

**Fig. 1:**
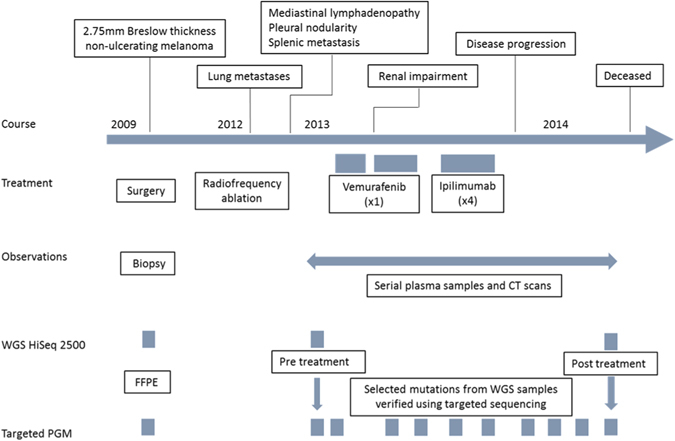
Outline of study design. Timeline indicates, when samples were collected, the type of sequencing that was undertaken, treatment administered and key stages in disease development

To characterise the impact of therapy on tumour genetic diversity, we performed WGS using cfDNA from samples taken before vemurafenib and after ipilimumab treatment, a formalin-fixed paraffin-embedded (FFPE) tumour biopsy and genomic DNA from peripheral blood mononuclear cells (Fig. 1).

**Fig. 2:**
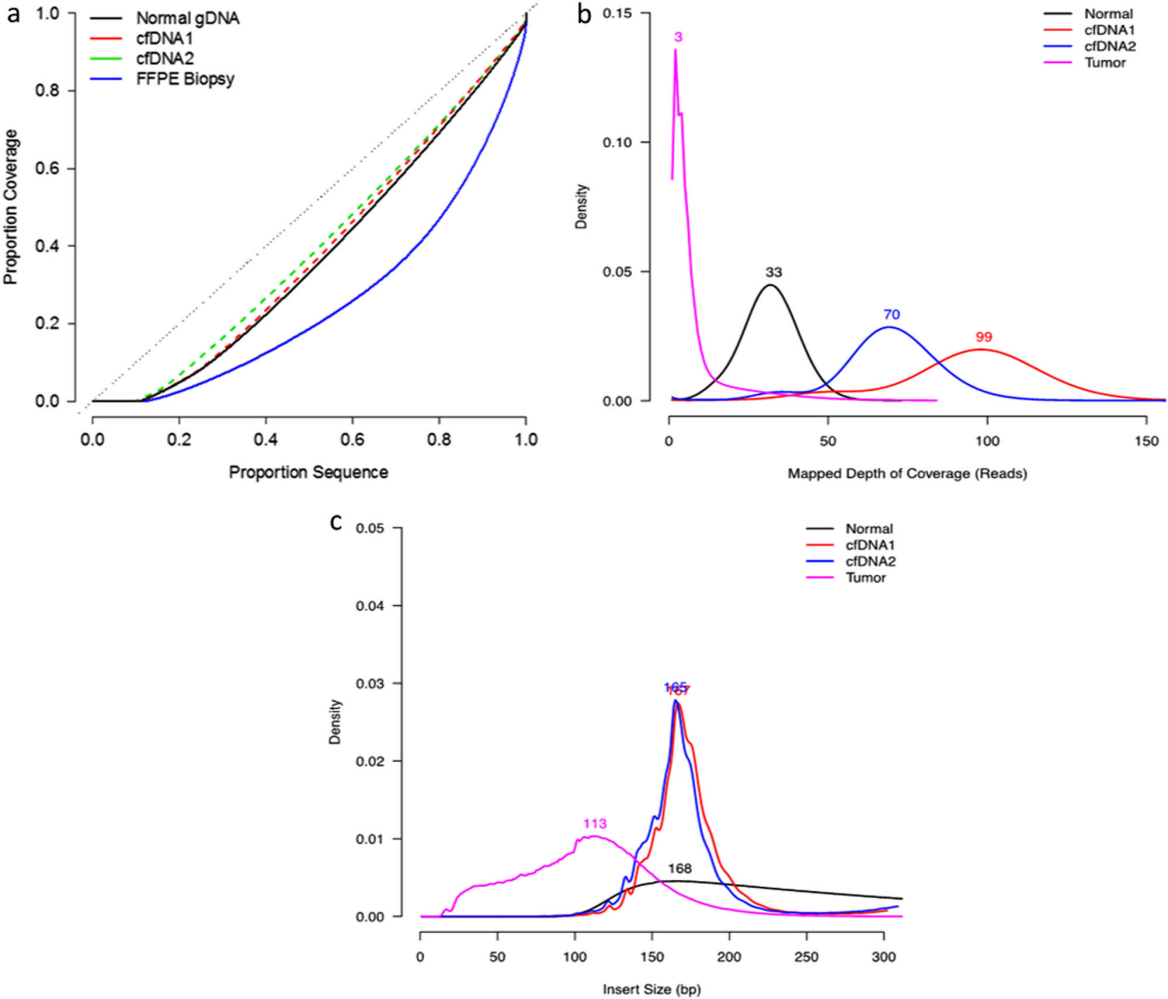
Coverage uniformity of WGS libraries. **a** Represented is the cumulative proportion of sequencing coverage per cumulative proportion of sequence in whole genome sequencing across normal germline DNA (gDNA) from peripheral blood mononuclear cells, two cfDNA time points, and an archival FFPE tissue biopsy from a metastatic melanoma patient. If coverage was perfectly uniform across the genome coverage the relationship would be linear with gradient one. **b** Mapped depth of coverage distribution for WGS sequencing runs. The range of the coverage distribution is truncated at 150. Each trace is annotated with the mode of the distribution. **c** Insert size distribution of sequencing reads for the sequencing runs. The distribution is truncated at 300 base pairs. Each trace is annotated with the mode of the distribution

To address the low abundance of ctDNA in cfDNA we developed a low input (50 ng) DNA library preparation method. Post-sequencing metrics showed similar depth of read uniformity and depth of coverage distribution (Fig. 2a and b) compared to libraries prepared from genomic DNA. As expected, insert sizes of ctDNA were small (Fig. 2c). The pre-therapy and post-therapy cfDNA samples were sequenced to an average depth of 100× and 71× respectively. Ninety-eight percent of the genomes were covered to a depth greater than 30×. The normal sample, obtained from peripheral blood mononuclear cells, was sequenced to a mean depth of coverage of 34×, with 96% of the genome covered 30× or more. The archival tumour tissue obtained from FFPE material performed poorly compared to the normal and cfDNA samples however, with much greater variability observed in coverage across the genome.

### Identification of somatic genomic alterations from cfDNA using WGS

WGS was applied to cfDNA extracted from the plasma sample taken at time point 1 and an optimised bioinformatics pipeline was developed and applied to detect somatic single nucleotide variants (SNVs). Briefly, to control sequencing artefacts, we developed a two stage somatic mutation calling algorithm. First, we identified candidate mutations using a full probabilistic model applied to sequencing alignments correcting for sequencing errors using empirical confusion matrices. Second, we applied de novo assembly to regions in which candidates were identified to guard against artefacts caused by genome divergence from the human reference genome sequence.

### Time point 1

After filtering candidate somatic mutations to control false positive rate (SOM), we detected 91 non-synonymous exonic SNVs by WGS of the cfDNA that ranged from 5–16% variant allele frequency (VAF) (Supplementary Table 1). Using an orthogonal custom-made Ion Torrent targeted deep sequencing panel (TSP) at an average depth greater than 3000× to cover the positions of the 91 SNVs, we were able to validate 84 somatic SNVs in this cfDNA sample. Not unexpectedly, the FFPE sample was of insufficient quality to yield accurate WGS data.^28, 29^ We therefore performed TSP sequencing on the tumour sample. The vast majority of mutant alleles detected by WGS of cfDNA were present in the primary tumour DNA (88/91), with VAFs in the primary tumour varying from 3 to 96%. Notably three variants were validated in the cfDNA that were not detected in the primary tumour DNA using TSP at 3000× (Supplementary Table 2). Conversely, seven variants detected in the biopsy were not seen in the WGS of the pre-treatment cfDNA sample. These were all subclonal with VAFs ranging from 4 to 33%. Where these discrepancies were observed and variants were not detected, only the major alleles were present as opposed to variants being present but not being called.

WGS of cfDNA allowed us to identify an activating mutation in proto-oncogene B-Raf (BRAF) (V600R) at 6% VAF, concordant with a VAF of 5% using TSP where the coverage was 131× and 7946×, respectively. No other exonic variants were detected that have previously been implicated in melanoma, although high-level mutations were detected in tumour suppressor genes (TRPS1 and CSMD1), pinpointing to their role as potential disease drivers in this patient. Importantly, using cfDNA WGS we were able to reveal non-coding mutations, including a pathogenic mutation in the TERT promoter that has been reported previously in melanoma.^30, 31^

### Time point 10

All of the exonic SNVs detected by WGS and verified using TSP1 in time point 1 were detected in time point 10, although in many instances there was considerable variation in the VAFs between the pre-treatment and post-treatment samples. Furthermore, all seven SNVs that were not detectable in cfDNA at time point 1 but were found in the biopsy were detectable in the cfDNA at time point 10.

Using WGS we also identified 121 exonic SNVs in the post-treatment sample that had not been detected in pre-treatment time point 1, with VAFs ranging between 5 and 23%. To investigate the properties of these variants, a second TSP panel was generated that targeted the 121 candidates. Ninety-nine percent (120) of the targeted candidate variants were confirmed in time point 10 cfDNA using the orthogonal TSP method and 93% (111) were also present in the tumour biopsy (Supplementary Table 3). One SNV (i.e., 1% of all validated cfDNA variants) in RP11-110A12.2.1 was found in the biopsy but not plasma time point 10, although it was detectable using TSP2 in earlier plasma time points. Ten SNVs (8%) were verified in the relapse cfDNA but were not present in the biopsy tumour sample (Supplementary Tables 3 and 4). Importantly, of these ten variants, five were detected in pre-treatment WGS from cfDNA. One of these variants occurred in INO80, a gene that is required for super-enhancer mediated oncogenic transcription. Furthermore it has been implicated in tumour growth in melanoma, where its protein expression is elevated.^32^

The pre-treatment time point 1 sample contained 96 of the 120 validated time point 10 SNVs. The majority of these were detectable at low levels, with VAFs typically below 5% and hence were likely below the limit of detection by WGS in time point 1. This suggests that sub-clones carrying these mutations were selected by the treatment. Aside from the exonic SNVs, the mutation in the promoter region of TERT was detectable at similar levels in time points 1 and 10 by WGS. Interestingly, due to the highly repetitive DNA sequence in this region and the short amplicons required in cfDNA panel design, it was not possible to design primers to verify this variant by targeted sequencing.

### Correlation between VAF and imaging

**Fig. 3:**
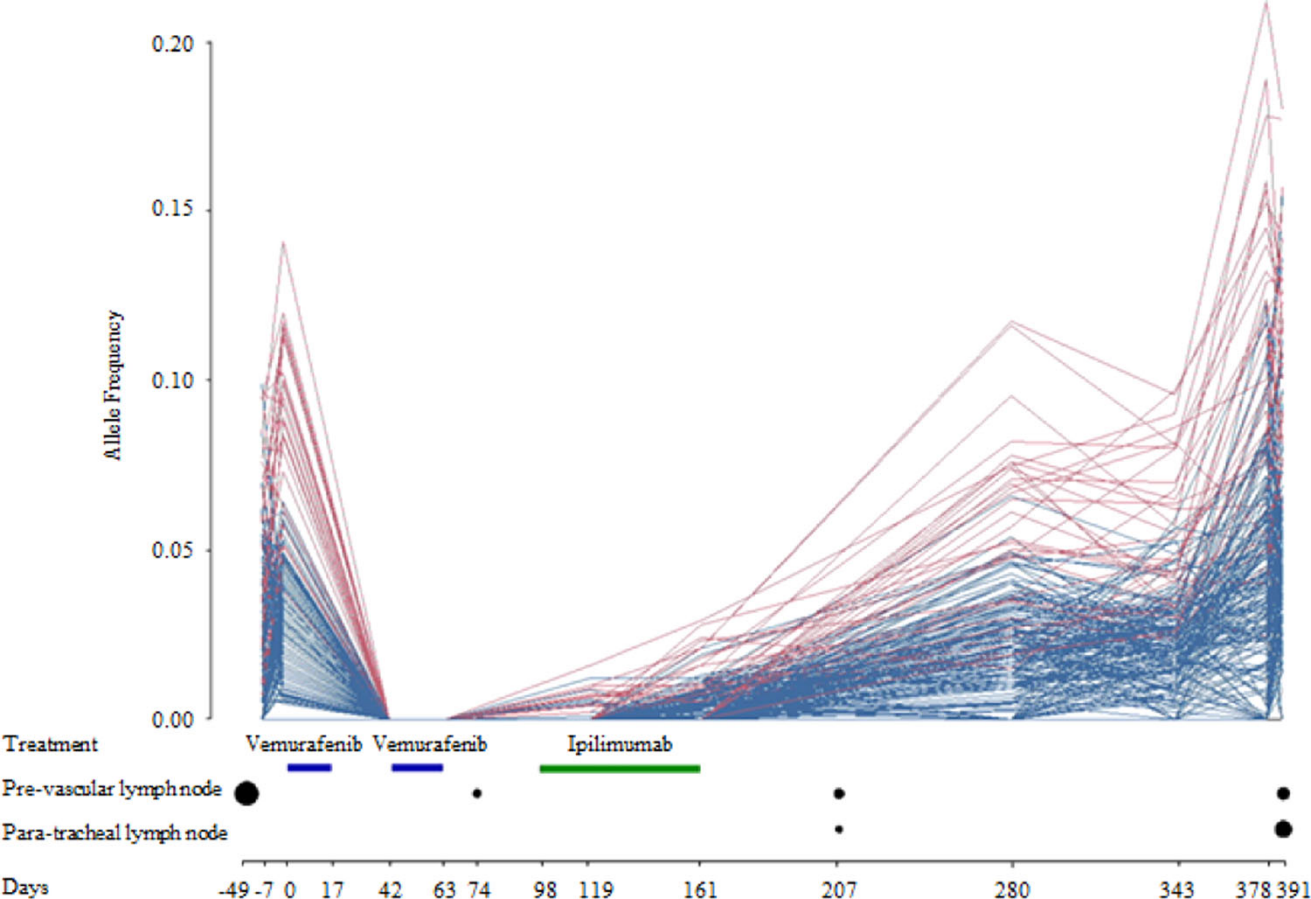
Mutational profile of variants detected by targeted sequencing. VAFs of candidate mutations detected by WGS were assessed at 10 time points starting 7 days before treatment (of these, two time points were obtained before treatment and the remaining eight during therapy). This data was analysed in tandem with CT scan imaging of the patient taken over the same time period. Estimated volumes from CT imaging are represented as circles scaled by relative proportion in size. Two different major subclones defined by hierarchical agglomerative clustering are highlighted in *blue* and *red*

In order to track quantitative changes in the mutation profile identified previously by WGS over time, we next used both the TSPs and compared this data with CT scans that were taken to monitor tumour size pre- and post-treatment on a three monthly basis (Fig. 3). In the two cfDNA samples sequenced prior to systemic treatment onset, the BRAF V600R variant, which is the only known driver mutation in melanoma detected in this patient, was consistently detected at similar levels of 5% VAF. Upon commencement with vemurafenib treatment, the BRAF V600R variant was no longer detectable by amplicon sequencing. CT imaging showed a decrease in tumour size in the same time period. Vemurafenib treatment was halted before disease progression because of toxicity and alternative therapy with ipilimumab was initiated. CT scans following completion of ipilimumab treatment showed an increase in tumour burden. This was reflected in BRAF V600R variant levels that increased from undetectable during vemurafenib treatment to 8% VAF at time point 10. A number of the exonic SNVs measured followed the BRAF V600R trajectory. Importantly, re-emergence of mutations (day 119) occurred nearly 3 months prior to clinical identification by imaging (day 207). Additionally hierarchical agglomerative clustering was used to group the mutations across all samples based on VAF values, with two major groups being identified in the data (Fig. 3). Separately the similarity between cfDNA and tumour samples based on their VAFs was evaluated (Supplementary Figure 1), with the latter having much higher VAFs than the plasma samples either taken pre-treatment / post relapse or during the period the patient responded to treatment.

### Mutational signature of melanoma

**Fig. 4:**
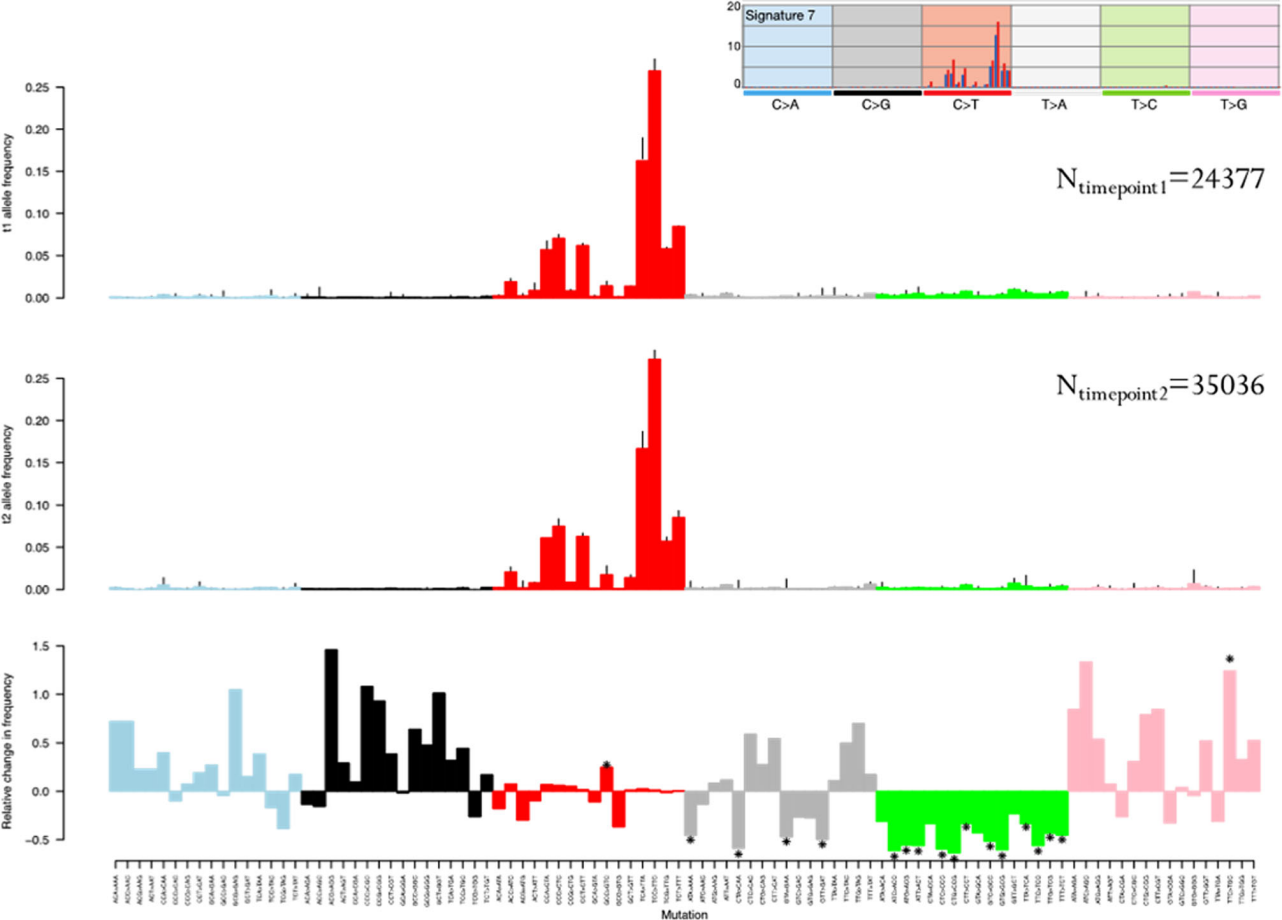
Melanoma mutation signature from cfDNA WGS. The *vertical axis* in panels 1 and 2 indicate the percentage of each mutation type in WGS time point 1 and WGS time point 10, respectively, with the type of mutations shown on the *horizontal axis*. The third panel depicts the relative changes in mutation type between the two time points

Finally, we evaluated the particular spectrum of mutations in this patient before and after treatment as large-scale analyses of cancer genomes have shown that distinct combinations of mutational signatures operate across cancer types.^33^ In WGS time point 1 and time point 10 a total of 24,377 and 35,036 variants, respectively, were identified, which is consistent with the high somatic mutation burden previously described in melanoma.^34^ Both WGS cfDNA samples also exhibit the classical mutation signature of C > T transition mutations based on the sequence context and notably, these mutations occur more frequently on the untranscribed strand than the transcribed strand.^33^ This is due to the formation of pyrimidine dimers that arise due to ultraviolet light exposure and is referred to as ultraviolet B -signature (Fig. 4). Furthermore, though VAFs correlate with increasing tumour burden across all sequence contexts, we observe a significant (*p* < 0.05, BH corrected FET) decrease in the number of A·T > C·G mutations compatible with sub-clonal pruning during treatment.

## Discussion

The genomic classification of tumours and the resulting opportunity for molecularly informed treatment is driving much innovation in oncology. The utility of genomics will be determined by understanding the relationships between acquired genetic alterations, the type of treatment administered, and the dynamics of mutations during and after treatment. For solid tumours, progress is limited by the difficulty of collecting (a) repeat invasive biopsies during treatment and (b) obtaining high quality tumour material. CfDNA sequencing on the other hand enables standardised, minimally invasive serial monitoring of tumour variants without the need for fresh tumour biopsies.

In this study, we analysed the cfDNA genome using minimal input DNA in a PCR-free approach, thus demonstrating the feasibility of WGS on low amounts of input DNA without the requirement for PCR amplification. This compares favourably to current approaches as it avoids preferential GC bias, which can greatly affect outcome data^35^ and gives greater confidence in accurate variant calling and quantification of variant allele frequencies. Moreover, detection of the TERT variant, which is one of the most common somatic variants in melanoma patients^30, 31, 36^ highlights one other key advantage of our PCR-free method compared to amplicon based approaches. Due to the highly fragmented nature of cfDNA, a prerequisite for primer design is that the amplified regions must be short, ideally ≤160 bp. This can make primer design extremely challenging especially for repetitive regions. Using targeted sequencing, it was not possible to design primers that specifically amplified the desired region of interest in the TERT promoter and this mutation would have been missed.

However, using a PCR-free approach also has potential disadvantages. Despite our optimisation of library preparation and bio-informatics pipelines, the DNA input requirements were still high at 50 ng. This means that our method on average requires at least 20–50 ml of peripheral blood.

An alternative to reduce the blood volume required would be to use reduced-error PCR by uniquely tagging individual cfDNA molecules. However not all PCR errors are removed via tagging, particularly if they are introduced in the first round of PCR. Additionally as cfDNA is at such a low concentration, greatly reducing the input may increase sequencing biases.

A further consideration is that the majority of ctDNA is derived from cells undergoing apoptosis but not all malignant melanocytes will experience this process, meaning ctDNA analysis may not give complete representation of the tumour. To our knowledge though, no studies have been published detailing the average lifespan of malignant melanocytes. However previous studies have shown changes in ctDNA levels in melanoma patients within 3 weeks of treatment commencing.^37^

The approach presented here may have utility in advanced cancers where median tumour DNA burden is high at 5–10 % ^17, 19, 38^ and patients are often too frail to undergo tissue biopsy. This indication currently represents the major focus for drug development and drug approval from pharmaceutical industry and regulators, respectively. Compared to targeted deep sequencing, our approach is less sensitive. Increasing the depth of sequencing is technically feasible and would overcome this issue, but is currently still prohibitive due to cost. To detect mutation frequencies f below 1%, the depth of coverage *X* would need to be scaled as *X* = κ/((α × f)) where alpha is the library efficiency and kappa is the read count scaling efficiency which is a function of PCR duplication rate and variation in coverage.

There is increasing evidence that some cancers exhibit intratumoral and intermetastatic heterogeneity that arises due to clonal and subclonal evolution. Tumour mutation status can differ between metastatic sites in the same patient.^1^ Several studies have highlighted how NGS technologies can be used to identify and track large numbers of somatic aberrations in parallel, thus revealing vital information regarding tumour evolution.^15, 24^ Here, we show that using WGS of cfDNA before therapy initiation we can confidently identify a subset of acquired mutations that were not present in the primary tumour biopsy using a deep sequencing approach, but that subsequently contributed to the relapse clone. The majority of putative mutations identified at relapse in WGS time point 2 that were not initially identified in WGS time point 1 were present in both biopsy and cfDNA at low level prior to treatment. We believe it is highly unlikely that the emergence of variants in WGS time point 2 that were not detectable in WGS time point 1 can be attributed to experimental variations, as the different samples were processed using exactly the same protocol. Also factors such as input cfDNA were kept the same to minimise any bias and post-sequencing metrics including depth of coverage were comparable. Separately, three relapse variants were not detectable in the biopsy but were present in WGS of pre-treatment cfDNA and verified by the orthogonal TSP approach, highlighting that the analysis of pre-treatment cfDNA by WGS reveals potential drivers of relapse early on in the disease course that would be missed by single biopsy sequencing due to spatial tumour heterogeneity. Furthermore, our data supports clonal selection as the major underlying mechanism for clonal evolution in this case and suggests that, contrary to chemotherapy, BRAF or checkpoint inhibitors are not mutagenic.

Importantly, thanks to the large number of somatic mutations detected by our genome-wide approach, we are able to detect the UV mutation signature in cfDNA. Future studies will be required to evaluate the clinical utility of measuring and tracking this and other signatures such as the smoking signature of lung cancer tumours in plasma samples from high-risk patients.^39^

Finally, using the cfDNA WGS results to inform the design of a targeted deep sequencing panel allowed us to track mutations while the patient was going through treatment with targeted therapy and immunotherapy. Interestingly, between days 280 and 343 the allele frequencies of one of the major subclones generally decreases, whilst this effect was not observed in the other major subclone. We believe it is unlikely that the decrease in VAFs is due to a systematic error as all samples for targeted sequencing were processed and analysed in exactly the same manner. Therefore it is feasible that the growth rate of the subclones differed, with one slightly shrinking over this time period. Tracking mutations allowed us to detect potential loss of therapy response nearly 3 months prior to a CT scan demonstrated disease progression on completion of ipilimumab treatment. A repeat biopsy was not taken at this time point to compare with the cfDNA analysis as treatment would not have been altered according to current clinical guidelines and it would have been difficult to obtain due to the distribution of metastatic disease. Although the clinical implications of early detection of molecular relapse must be further evaluated in clinical trials, we can speculate that ipilimumab might have been stopped earlier as cfDNA showed an increase in the frequency of plasma mutations. The cost savings associated with early discontinuation of therapy far outweigh the cost of WGS sequencing in this situation. Conversely, if the patient had responded well to therapy, use of plasma DNA monitoring may have abrogated the need for regular CT scans with associated radiation exposure. Ipilimumab monotherapy has now mostly been replaced with PD-1 inhibitor treatment, either in combination with ipilimumab (if patients are fit enough), or alone (for less fit patients).^40^ The role plasma DNA monitoring may play in this setting again requires further evaluation, although with potential for greater utility as currently PD-1 inhibitor treatment generally continues every 2 or 3 weeks until radiological disease progression, as assessed by 3 monthly CT scans.

In conclusion, we show that WGS can be carried out on cfDNA using a PCR free protocol with low input DNA and that bioinformatics tools can be developed to accurately detect variants with high sensitivity. We reliably detect clinically relevant coding and non-coding mutations and were able to identify UV exposure as the disease causing mechanism from plasma. Combining our approach with targeted deep sequencing enabled us to describe the effects of targeted treatment with vemurafenib and ipilimumab on mutations detected by WGS from cfDNA. We establish that the re-emergence of mutations correlates with clinical disease progression. Finally, we propose that, provided costs of sequencing reduces, our approach could be used to systematically evaluate the clinical utility of cfDNA for guiding clinical decision making in clinical trials.

## Methods

### Ethical approval

The National Health Service (NHS) Health Research Authority South Central—Oxford C Research Ethics Committee approved this study and all research was performed in accordance with relevant regulations and guidelines. Written informed consent was obtained from the patient according to current Oxford Radcliffe Biobank (ORB) guidelines (Oxford C Research Ethics Committee No: 09/H0606/5+5).

### Study design

This case study was of an 84-year-old male patient with malignant melanoma. The patient’s clinical treatment had five phases: detection of primary tumour, detection of metastases, molecularly targeted treatment, immunotherapy, and progression.

Detection of primary tumour. In October 2009 the patient presented with a 2.75 mm Breslow thickness non-ulcerating melanoma on his left ear. The melanoma was excised and preserved using formalin-fixation and paraffin-embedding (SOM).

Detection of metastases. In December 2012 lung metastases were detected, which were initially treated with radiofrequency ablation. Systemic treatment was held off while the patient was asymptomatic. Four months after ablation, further mediastinal lymphadenopathy plus pleural and splenic metastases were detected by CT scan.

Molecularly targeted treatment. Following detection of further metastases the patient’s archival primary tumour biopsy tissue was screened for targetable mutations using a limited in-house amplicon sequencing panel targeting known cancer driver mutations. Amplicon sequencing detected a rare (c. 5% ^41^) BRAF V600R mutation in the melanoma, predicted to be sensitive to the BRAF inhibitor vemurafenib.^42^ In May 2013 the patient started treatment with vemurafenib 960 mg twice daily continuous dosing, with radiological response. 17 days after initiation, the patient was admitted with vomiting and worsening renal function. Treatment was suspended for 25 days and then recommenced at 480 mg twice daily 2 weeks on, 2 weeks off. Due to recurrent renal impairment vemurafenib treatment was discontinued permanently on 8th July 2013. Despite intermitted treatment, imaging indicated partial response with reduction in a prevascular lymph node from 43 to 16 mm.

Immunotherapy. After a break of 39 days, second-line treatment with the CTLA-4 inhibitor ipilimumab was commenced on 15 August 2013, with completion of four treatment cycles by 17th October 2013.

Progression. Radiological imaging indicated disease burden increase 4 weeks after ipilimumab treatment was completed. Further disease progression was confirmed on CT scans in December 2013 and June 2014, with the patient succumbing to disease in the Autumn of 2014.

### Clinical sample collection

Between May 2013 and June 2014 serial blood samples were collected in ethylenediamine tetraacetic acid tubes before, during and after treatment with vemurafenib and ipilimumab. 20–50 mls were collected across 10 time points and processed within 4 h of blood draw. Blood was centrifuged for 10 min at 3000 rpm to separate the plasma. The plasma was subsequently transferred to fresh microfuge tubes and centrifuged for 10 min at 7000 r.p.m. The supernatant was stored in aliquots at −80 °C until DNA extraction.

### DNA extraction

CfDNA was extracted using the QIAamp circulating nucleic acid kit (Qiagen), according to the manufacturer’s instructions. Input plasma volumes ranged from 5 ml to 20 ml per time point, in 5 ml batches. DNA was first eluted into 30 µl of buffer AVE and subsequently a second elution of 30 µl to maximise yield. Samples eluted into separate tubes from the same time point were pooled and stored at −20 °C. Ger﻿mline DNA (﻿GDNA) was extracted from the normal peripheral blood leucocytes using the QIAamp DNA mini kit (Qiagen), in accordance with the manufacturer’s instructions. Matched tumour DNA (tDNA) was extracted from FFPE archival tumour material. Tumour area was marked out on ten 5 µM thick unstained sections and macro-dissection carried out to enrich for tumour material. TDNA was extracted using the QIAamp FFPE DNA extraction kit (Qiagen) and eluted into 20 µl buffer EB. All cfDNA and tDNA samples were quantified on a Qubit fluorometer (Invitrogen) using the high sensitivity dsDNA kit and gDNA was quantified using the broad range dsDNA kit.

### Whole genome sequencing

Libraries from gDNA, cfDNA and tumour specimens were prepared using the TruSeq Nano LT DNA Sample Preparation Kit (Illumina Inc) 350 bp insert protocol according to the manufacturer’s instructions, with some exceptions. As cfDNA is already highly fragmented, no further fragmentation of the samples was carried out. For each sample 50 ng of input DNA was used and the samples did not undergo any PCR amplification steps. Additionally the clean-up steps to remove small and large fragments were omitted. Final libraries were eluted in a volume of 22 µl resuspension buffer. Fragment distribution pre- and post-library preparation was determined by running 1 µl aliquots on a bioanalyzer 2100 (Agilent Technologies). Libraries were diluted 1:500 and quantified in triplicate by qPCR using the Kapa quantification kit (Kapa Biosystems) on an ABI 7500 (Life Technologies). Based on these quantifications, libraries were diluted to 2 nM and the final input concentration was 10 pM. DNA libraries were clustered onto Flow Cells using the c-Bot v3 kit, and sequencing with paired end reads was carried out on a HiSeq 2500 using v3 chemistry in High Throughput mode.

### Targeted resequencing

Custom panels were designed to confirm the presence of SNVs identified by WGS in the cfDNA samples. Candidate chromosome positions based on the hg 19 human genome reference sequence were uploaded to Ion Ampliseq^TM^ Designer version 3.0.1 and the DNA type selected ‘FFPE’, with all target amplicons being ≤175 bp. Sequencing was carried out on the Ion Torrent Personal Genome Machine (PGM, Life Technologies). Briefly, a multiplex PCR was performed using the Ion Ampliseq 2.0 kit, where 5 ng input DNA was used and amplified using 22 PCR cycles. After PCR, primer sequences were partially digested with FuPa and samples were indexed using Ion Xpress adaptors according to the manufacturer’s instructions (Life Technologies). Libraries were purified using the Axprep Mag PCR clean up kit (Axygen Biosciences) and then diluted 1:200 prior to quantification by qPCR on an ABI 7500 using the Ion Quantitation Kit (Life Technologies). Libraries were diluted to a 20 pM concentration and were pooled. Template positive ISPs were generated containing clonally amplified ISPs using the OneTouch 2 200 template kit v2 (Life Technologies). Samples were enriched on the Ion OneTouch ES and subsequently sequenced using 316 or 318 chips on the PGM using the Ion Sequencing 200 bp chemistry (Life Technologies).

### Bioinformatics analysis

From the WGS data tumour-specific variants were identified through a combination of alignment-based and de novo assembly-based methods. Candidate variant positions in the cfDNA alignment were defined as positions with at least three high-quality non-genomic alleles and positions with unexpectedly high or low total coverage were ignored. For each candidate position, a range of frequentist and Bayesian statistics were computed, measuring the strength of evidence for the presence of non-genomic alleles. All statistics incorporate empirical confusion matrices for all binary alignment map ﻿(BAM) files involved. For each candidate position, a variant background haplotype was heuristically constructed, and the presence of this haplotype was verified in the cfDNA BAM and its absence in the genomic BAM using kmer-based statistics.

Sequence data from the PGM was analysed using the combined output of Strelka v1.0.15 and MuTect2. Target regions were defined using the relevant bed files for the two custom panels. The positions of all candidate somatic variants were investigated in Integrated Genomics Viewer using BAM files aligned to the hg19 human genome reference sequence. For further information on bioinformatics analysis: see Supplementary Methods.

### Code availability

The code is currently not readily available as the authors are working on a separate manuscript for the software package.

### Data availability

Sequence data has been deposited at the Sequence Read Archive (SRA) database (https://www.ncbi.nlm.nih.gov/sra/), under accession number SRP111727.

## Electronic supplementary material

Supplementary Methods

Supplementary Figure 1

Supplementary Table 1

Supplementary Table 2

Supplementary Table 3

Supplementary Table 4
