# Supplementary material for: Characterisation of the changing genomic landscape of metastatic melanoma using cell free DNA
Source: NPJ Genom Med. 2017 Sep 4;2:25. doi: 10.1038/s41525-017-0030-7 (PMC5654504; doi:10.1038/s41525-017-0030-7)
Supplement: Supplementary file 1 — Supplementary Methods [file 41525_2017_30_MOESM1_ESM.docx]

# **Supplementary Methods**

# **Variant calling from cfDNA**

## **Calling strategy – High-level overview**

We employ a two-stage calling strategy: first, we identify candidate variants using a frequentist and a Bayesian test. The frequentist test is designed for positions that are homozygous in the germline genome and treats each allele present in the germline BAM, independent of its germline frequency, as non-somatic. The Bayesian test, in contrast, is based on a full genotype model for the germline and the tumour genome, and can be used for variant calling at germline-heterozygous positions.

We independently use both tests to identify candidate variant alleles and their positions, and feed the putative variants through a “de novo” filter. The “de novo” filter uses a de Bruijn graph approach (as in *de novo* assembly) to eliminate variant calls that might result from systematic differences in alignment (e.g. due to different fragment sizes) or alignment ambiguity. The rationale behind this procedure is to benefit from the alignment-independent robustness of *de novo* approaches, without having to rely on graph-topological operations to *identify* variants.

Both the frequentist and the Bayesian test employ empirical confusion matrices, which we use to model the probability of sequencing errors, conditional on base quality and within-read position. The confusion matrices are populated under the assumption that the large majority of germline-homozygous positions will also be homozygous in the tumour genome (even in the most strongly mutated tumours, per-base mutation rates are < 1/10,000), and that we can use these positions to characterize the properties of the sequencing error process. As error profiles can vary systematically between sequencing runs and samples, we compute a separate confusion matrix for each input BAM.

The calling strategy described here assumes the availability of germline as well as cfDNA/tumour sequencing data; although the strategy could, in principle, be adapted to work in a cfDNA/tumour-only context, the underlying statistical models would have to be modified.

## **Computation of BAM-specific confusion matrices**

To account for systematic differences in error rate between samples, we compute an empirical confusion matrix for each input BAM file. Iterating over all positions of the reference genome, we extract the alleles mapped to each position. We ignore all positions for which total column coverage shows strong evidence for deviation from the BAM’s mean coverage (two-sided Poisson p-value with expected value equal to BAM mean coverage < 1e-6), all columns with majority allele length > 1 bases, and all columns which might be germline-polymorphic (see below). For all remaining columns, we assume that the “true” allele is identical to the column’s majority allele, and all non-majority alleles are counted as “erroneous”, keeping track of base quality and within-read position of the base.

These matrices give an empirical estimate of the probability that a base specified by a sequencing read is a sequencing error, conditional on the source BAM, the base’s specified quality and the within-read position of the base. We note that these estimates are conservative (i.e., they slightly over-estimate error rates), for they include non-erroneous alleles deriving from low-frequency somatic mutations.

Identification of germline-polymorphic positions: We compute the likelihood of the observed column data under the two models

- Model M1 (“homozyzgous with 1% error”): c_Major_ ~ Poisson(0.99 x C_avg_), c_Minor_ ~ Poisson(0.01 x C_avg_)
- Model M2 (“germline heterozygous”): c_Major_ ~ Poisson(0.5 x C_avg_), c_Minor_ ~ Poisson(0.5 x C_avg_)

, where C_avg_ is the BAM’s average coverage, c_Major_ is the column count of the column majority allele, and c_Minor_ is the column count of all non-majority alleles. If, assuming uniform priors on the models, P(M1)/[P(M1)+P(M2)] ≤ 0.99, we classify the column as “potentially germline-polymorphic”.

## **Frequentist test for somatic variation**

We test the null hypothesis that potentially variant, non-germline alleles at a position in the tumour/cfDNA BAM are due to sequencing errors at that position, assuming that the expected number of sequencing errors follows from the BAM’s empirical confusion matrix (taking into account base qualities and within-read position, see above). Depending on the number of expected errors, we use either a Χ^2^-Goodness-of-fit (expected errors >= 10) or a binomial test, and report the p-value of the observed data (observed vs expected allele counts). If the number of observed variant alleles is smaller than the expected number of such alleles, we always return a p-value of 1.

We only apply the test to positions with non-abnormal coverage patterns (using the same Poisson test and p-value-threshold as defined above), and only to positions that were not classified as “potentially polymorphic” in the germline BAM (see above). For conservativeness, we only apply the test to alleles that are strictly not present in the corresponding column of the germline BAM, and each potentially variant allele is tested separately (that is, when testing allele *x*, all non-*x* alleles are treated as non-variant alleles). We only use reads with mapping quality >= 10. To speed up computation, we only consider positions with at least one potential variant allele with coverage >= 3.

## **Bayesian test for somatic variation**

We compare the posterior probabilities of the following four models (uniform prior over models; using the Laplace approximation to evaluate the posterior probability integral):

- Model 1: Germline genome homozygous, no tumour admixture
- Model 2: Germline genome heterozygous, no tumour admixture
- Model 3: Germline genome homozygous, tumour admixture with additional allele *a* at frequency *f*
- Model 4: Germline genome heterozygous, tumour admixture with additional allele *a* at frequency *f*

The total probability for the presence of somatic allele *a* is [ P(Model 3) + P(Model 4) ], and we define the Bayes Factor BF in favour of the presence of a as [ P(Model 3) + P(Model 4) ]/ [ P(Model 1) + P(Model 2) ].

For Models 1/3, we fix the germline genotype as the most common allele in the germline BAM; for Models 2/4, we use the two most common alleles from the germline BAM as germline genotype.

We use a uniform prior over alleles *a* considered at each position and a Gamma distribution (parameters 2, 2) prior for *f*. As in the frequentist case, we only consider variant alleles that are present at coverage >= 3 and positions with coverage within the acceptance region of the Poisson test (see above).

For the data observed in the cfDNA and germline BAMs (i.e. the corresponding columns in the two BAMs and the sequencing read alleles there-specified), we use a genotype likelihood model [^1^](file:///C:\Users\DILTHE~1\AppData\Local\Temp\1\Variant%20calling%20and%20ssDNA%20analysis.docx#_ENREF_1)^,^[^2^](file:///C:\Users\DILTHE~1\AppData\Local\Temp\1\Variant%20calling%20and%20ssDNA%20analysis.docx#_ENREF_2) conditional on allele frequencies specified by the considered model, employing the BAM-specific confusion matrices to model the probability of a sequencing error.

## **De novo filtering**

We construct a coloured de Bruijn graph with k-mer size k = 21 from the cfDNA/tumour (colour 1) and germline (colour 2) sequencing data.

For each candidate variant, we attempt to reconstruct the local haplotype (k – 1 bases in either direction) that the variant occurs on: we extract the reads carrying the variant allele from the cfDNA/tumour BAM and compute the consensus sequence of the 2k – 2 bases surrounding the variant (simple majority vote). The reconstructed local haplotype has length (2k – 2 + length of the variant allele). The variant passes the “de novo” filter if all k-mers from the local haplotype are present in the colour 1 graph and not present in the colour 2 graph.

In some instances, local haplotype reconstruction might fail. Therefore, we also construct a putative local haplotype in a reference-guided manner by inserting the variant allele into the surrounding 2k – 2 bases of reference sequence. The variant passes the “de novo” filter if all k-mers from the putative haplotype are present in the colour 1 graph; none of the k-mers from the putative haplotype are present in the colour 2 graph; and, finally, all of the k-mers from the corresponding reference-only haplotype (i.e. without the variant allele) are present in the colour 2 graph.

## **Thresholds**

We reported variants with (frequentist p ≤ 1e-6 OR BF ≥ 1e6) AND “passed de novo”.

## **Bibliography**

43. DePristo, M.A. *et al.* A framework for variation discovery and genotyping using next-generation DNA sequencing data. *Nat Genet* **43**, 491-8 (2011).

44. Li, H. A statistical framework for SNP calling, mutation discovery, association mapping and population genetical parameter estimation from sequencing data. *Bioinformatics* **27**, 2987-93 (2011).

45. Iqbal, Z., Caccamo, M., Turner, I., Flicek, P. & McVean, G. De novo assembly and genotyping of variants using colored de Bruijn graphs. *Nat Genet* **44**, 226-32 (2012).
