# Supplementary figures and images for: Characterisation of the changing genomic landscape of metastatic melanoma using cell free DNA
Source: NPJ Genom Med. 2017 Sep 4;2:25. doi: 10.1038/s41525-017-0030-7 (PMC5654504; doi:10.1038/s41525-017-0030-7)

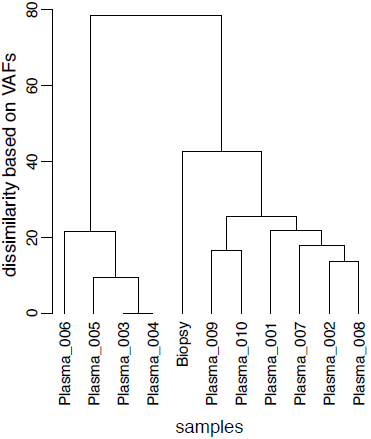


**Supplementary Figure 1.** Disimilarity between tumour and plasma samples based on VAF values.

Supplement: Supplementary file 2 — Supplementary Figure 1 [file 41525_2017_30_MOESM2_ESM.docx]
